# Reliability of the MOCART (Magnetic Resonance Observation of Cartilage Repair Tissue) 2.0 knee score for different cartilage repair techniques—a retrospective observational study

**DOI:** 10.1007/s00330-021-07688-1

**Published:** 2021-01-29

**Authors:** Markus M. Schreiner, Marcus Raudner, Sebastian Röhrich, Martin Zalaudek, Michael Weber, Georg Kaiser, Silke Aldrian, Catharina Chiari, Reinhard Windhager, Siegfried Trattnig

**Affiliations:** 1 Department of Orthopedics and Trauma Surgery, Medical University of Vienna, Währinger Gürtel 18-20, 1090 Vienna, Austria; 2 High Field MR Centre, Department of Biomedical Imaging and Image-guided Therapy, Medical University of Vienna, Vienna, Austria; 3 Austrian Cluster for Tissue Regeneration, Vienna, Austria; 4 Christian Doppler Laboratory for Clinical Molecular MR Imaging (MOLIMA), Department of Biomedical Imaging and Image-guided Therapy, Medical University of Vienna, Vienna, Austria

**Keywords:** Cartilage, Knee, Magnetic resonance imaging

## Abstract

**Objectives:**

To evaluate the reliability of the MOCART 2.0 knee score in the radiological assessment of repair tissue after different cartilage repair procedures.

**Methods:**

A total of 114 patients (34 females) who underwent cartilage repair of a femoral cartilage lesion with at least one postoperative MRI examination were selected, and one random postoperative MRI examination was retrospectively included. Mean age was 32.5 ± 9.6 years at time of surgery. Overall, 66 chondral and 48 osteochondral lesions were included in the study. Forty-eight patients were treated with autologous chondrocyte implantation (ACI), 27 via osteochondral autologous transplantation, five using an osteochondral scaffold, and 34 underwent microfracture (MFX). The original MOCART and MOCART 2.0 knee scores were assessed by two independent readers. After a minimum 4-week interval, both readers performed a second reading of both scores. Inter- and intrarater reliabilities were assessed using intraclass correlation coefficients (ICCs).

**Results:**

The MOCART 2.0 knee score showed higher interrater reliability than the original MOCART score with an ICC of 0.875 versus 0.759, ranging from 0.863 in the MFX group to 0.878 in the ACI group. Intrarater reliability was good with an overall ICC of 0.860 and 0.866, respectively. Overall, interrater reliability was higher for osteochondral lesions than for chondral lesions, with ICCs of 0.906 versus 0.786.

**Conclusions:**

The MOCART 2.0 knee score enables the assessment of cartilage repair tissue after different cartilage repair techniques (ACI, osteochondral repair techniques, MFX), as well as for different lesion types with good intra- and interrater reliability.

**Key Points:**

• *The MOCART 2.0 knee score provides improved intra- and interrater reliability when compared to the original MOCART score.*

• *The MOCART 2.0 knee score enables the assessment of cartilage repair tissue after different cartilage repair techniques (ACI, osteochondral repair techniques, MFX) with similarly good intra- and interrater reliability.*

• *The assessment of osteochondral lesions demonstrated better intra- and interrater reliability than the assessment of chondral lesions in this study.*

## Introduction

Injuries of the articular cartilage of the knee joint are common and remain challenging due to the avascular nature of articular cartilage and its limited regenerative potential [1–3]. Left untreated, symptomatic cartilage defects impair the quality of life and put affected patients at risk for the development of secondary osteoarthritis [4]. As a result, a variety of different surgical cartilage repair techniques have been developed over the last few decades with differing indications, outcomes, and associated costs.

Treatment options include bone marrow stimulation (BMS) such as microfracture (MFX) [5] and Pridie drilling [6], osteochondral repair techniques such as osteochondral autologous transplantation (OAT) [7], or acellular osteochondral scaffolds [8, 9] and cell-based repair techniques with various generations of autologous chondrocyte implantation (ACI) [10].

Even though arthroscopy remains the standard of reference for the evaluation of cartilage defects with the Outerbridge Classification [11] and the International Cartilage Repair Society Score [12], due to its invasiveness, it is rarely indicated.

Magnetic resonance imaging (MRI), however, remains the gold standard for the non-invasive assessment of articular cartilage and subchondral bone. However, meta-analyses show incoherent findings regarding the correlation of postoperative MRI examinations with clinical outcome [13–15].

In an effort to standardize the assessment after cartilage repair, the Magnetic Resonance Observation of Cartilage Repair Tissue (MOCART) [16] score has been introduced to facilitate reproducible, longitudinal assessments and comparability across studies.

The original MOCART score was already used for follow-up studies investigating OAT [17], ACI [18], and MFX [19] as well as in prospective studies that compared different repair techniques [20, 21].

To encompass the technological advancements and developments regarding surgical treatment options of cartilage defects of the knee joint and magnetic resonance imaging technology since the publication of the original MOCART score 15 years ago, the MOCART 2.0 knee score [22] has been recently introduced. In addition to the elimination of the variables “subchondral lamina,” “effusion,” and “adhesion,” the variable “subchondral bone” was renamed “subchondral changes” and the variable “bony defect or bony overgrowth” was introduced. To increase reproducibility and comparability across trials, a color-coded atlas depicting all variables of the MOCART 2.0 knee score was established as well. For expert readers, the MOCART 2.0 knee score demonstrated an almost perfect overall interrater (ICC = 0.84, *p* < 0.001) as well as intrarater (ICC = 0.88, *p* < 0.001) reliability. Access to the aforementioned atlas improved the overall interrater reliability of inexperienced readers from poor (ICC = 0.34, *p* < 0.019) to moderate (ICC = 0.59, *p* = 0.001) [22]. However, until now, the reliability has been assessed only for patients after ACI. Hence, it remains unclear whether the MOCART 2.0 knee score allows for the postoperative assessment after different cartilage repair techniques with a comparable interrater and intrarater agreement. Furthermore, it has not yet been evaluated whether the lesion type (chondral versus osteochondral) influences the applicability or the reproducibility of the MOCART 2.0 knee score.

Therefore, the aim of this study was to (a) evaluate the reliability of the MOCART 2.0 knee score in the postoperative assessment of patients after different surgical cartilage repair techniques; (b) compare it to the intrarater and interrater reliability of the original MOCART score; and (c) assess whether the intra- and interrater reliability differs between different surgical repair techniques and between patients with chondral and osteochondral defects.

## Materials and methods

This retrospective, single-center study was approved by the Institutional Review Board. Patients who underwent surgical cartilage repair of a femoral cartilage lesion in the knee joint and received at least one MRI follow-up examination at a 3-T system at our institution were selected and one postoperative MRI examination per patient was retrospectively included in the study. Selection of the follow-up time was random by drawing of lots to offer a representative distribution of a mixed patient cohort in terms of cartilage maturation. Patients were allocated to three different types of repair groups: an ACI group; an osteochondral repair technique group, which included patients after OAT or MaioRegen^®^ (Fin-ceramica) implantation; and an MFX group.

ACI was performed as a two-step procedure. In the first procedure, a cartilage biopsy was obtained arthroscopically from a non-weight-bearing area of the knee. After cell extraction, cells were cultivated and subsequently transferred onto a scaffold. For the second procedure of ACI, a mini-arthrotomy was used as a surgical approach. First, debridement of the cartilage defect to the subchondral bone was performed. In case of osteochondral lesions, additional bone grafting was performed using autograft spongiosa cylinders that were harvested from the iliac crest or the ipsilateral tibia using an OATS harvester; then, the cell matrix implants were cut to size, implanted, and held in place using fibrin glue.

OAT was performed as a single-step procedure for smaller lesions. Autograft spongiosa cylinders were harvested from the trochlea using an OATS harvester (OATS^®^, Arthrex) and transferred into the defect.

Microfracture (MFX) was performed arthroscopically after debridement and the establishment of stable cartilage shoulders using ChondroPick^®^ (Arthrex) as a one-step procedure as well [23].

### Magnetic resonance imaging

All imaging studies were conducted on 3-T MR systems (MAGNETOM Tim Trio, MAGNETOM Verio, MAGNETOM Prisma, Siemens Healthineers) using a dedicated eight-channel or 15-channel knee coil. The evaluated imaging studies were part of the clinical routine follow-up. Therefore, the parameters differed slightly between patients.

An exemplary routine MRI protocol for knee cartilage assessment after cartilage repair in the femoral condyle is presented in Table 1. The protocol included a three-dimensional localizer followed by a sagittal non-fat-saturated high-resolution proton-density-weighted turbo spin-echo (sag PDw TSE) sequence; a sagittal fat-saturated (Fs) PDw TSE sequence; a sagittal T1-weighted (T1w) TSE; and a coronal Fs PDw TSE sequence. For patients who underwent cartilage repair of the patellofemoral joint, the imaging protocol is to be complemented with an axial Fs PDw TSE sequence [24].

**Table 1:** Exemplary MRI protocol that fulfills the recommended requirements in terms of sequences and resolution for adequate assessment of the MOCART 2.0 knee score at 3 T

	Example parameters for a 3-T protocol			
	Sag PDw TSE 2 mm	Sag PDw TSE Fs	Sag T1w TSE	Cor PDw TSE Fs
Coil	8-channel knee	8-channel knee	8-channel knee	8-channel knee
TE (ms)	37	42	12	27
TR (ms) ± deviation	2000 ± 10%	3090 ± 10%	600	4250
Flip angle	90 ± 10%	90 ± 10%	90	180
Fat suppression	No	Yes	No	Yes
FOV (mm)	120	160	150	150
RFOV (%)	100	100	100	100
Acq. matrix	384	384	448	384
Scan (%)	85	100	100	100
Slices	19	25	25	25
Slice thickness	2	3	3	3
Interslice gap (%)	10	20	20	10
Slice orientation	Sagittal	Sagittal	Sagittal	Coronal
Acquisition time (TA)	03:20	04:06	02:48	03:29

### Image analysis

Image analysis was performed on a picture archiving and communication system (PACS) workstation (IMPAX EE R20, Agfa Healthcare N.V.) by an orthopedic resident (reader 1) and a radiology resident (reader 2), each with 4 years of experience in musculoskeletal MR imaging studies.

For the assessment of the MOCART 2.0 knee score, both readers had access to the atlas, which was published as supplemental material alongside the MOCART 2.0 knee score [22].

Imaging studies were assessed under supervision of the study coordinator in random order, and both readers were completely blinded to all patient details. First, both readers assessed the original MOCART score as well as the MOCART 2.0 knee score for a training dataset of twenty imaging studies that were not included into the study. After a 1-week interval, both readers assessed the original MOCART score for all patients of the study cohort. After a 4-week interval, to diminish recall bias, both readers assessed the MOCART 2.0 knee score for all patients. After another minimum interval of 4 weeks, readers assessed the MOCART 2.0 knee score a second time to allow for the assessment of intrarater reliability of the MOCART 2.0 knee score. After another minimum interval of 4 weeks, both readers assessed the original MOCART score a second time to allow for the assessment of intrarater reliability of the original MOCART score. Both readers did not receive feedback on their scorings between the different readings.

### Statistical analysis

All statistical calculations were performed using IBM SPSS Statistics for Windows version 25 (IBM). Continuous data are described using mean ± standard deviation. Descriptive statistics in addition to univariate ANOVA were used to compare defect size, time to follow-up, and age at examination between treatment groups, with Bonferroni’s correction for comparison of more than two groups. Linear-weighted kappa statistics and their 95% CI were calculated as an index for inter- and intrarater reliability of each ordinal scoring domain of the original MOCART score and the MOCART 2.0 knee score. Weighted kappa statistics were interpreted according to the criteria of Landis and Koch [25]. A kappa value of ≤ 0.20 indicated poor agreement, a kappa value of 0.21–0.40 indicated fair agreement, a kappa value of 0.41–0.60 indicated moderate agreement, a kappa value of 0.61–0.80 indicated substantial agreement, and a kappa value of ≥ 0.81 indicated almost perfect agreement.

Two-way mixed, absolute agreement, single-measure intraclass correlation coefficients (ICCs), and their 95% confidence intervals (95% CI) were calculated as an index of intra- and interrater reliability of the total resultant continuous MOCART and MOCART 2.0 knee scores.

ICCs were interpreted according to Koo and Li [26].

An ICC of < 0.50 indicated poor agreement, an ICC of 0.50–0.75 moderate agreement, an ICC of 0.75–0.90 good agreement, and an ICC > 0.90 excellent agreement.

*p* values equal or below 0.05 are considered to indicate statistically significant results.

## Results

### Patients

**Fig. 1:**
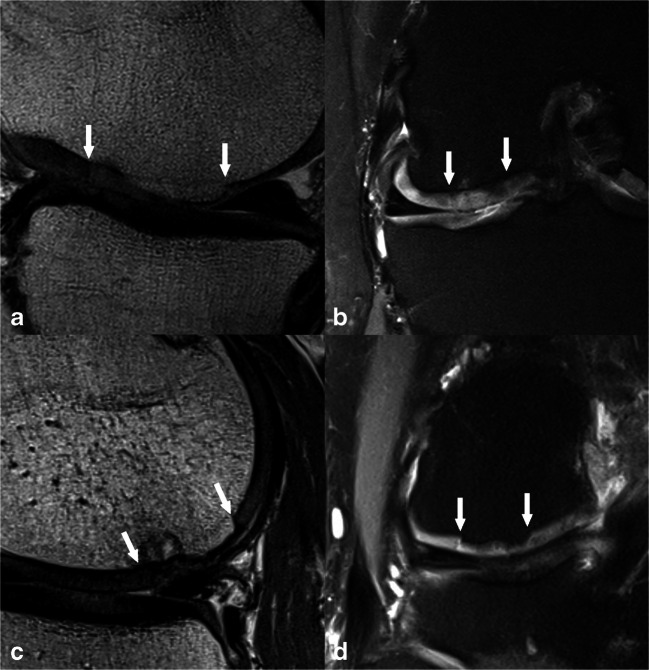
**a**, **b** A 42-year-old male patient 8 years after ACI with a major hypertrophic filling, complete integration, an intact surface, homogeneous structure, minor hyperintense signal intensity (anteriorly), a bony overgrowth ≥ 50% of adjacent cartilage thickness, and no subchondral changes, with a resultant MOCART 2.0 knee score of 80 points. **c**, **d** A 46-year-old male patient 6 years after ACI with minor hypertrophy, complete integration, an irregular surface < 50% of the tissue diameter, a homogeneous structure, minor hyperintense signal, a bony overgrowth ≥ 50% of adjacent cartilage, and a subchondral cyst of less than 5 mm diameter, which resulted in a MOCART 2.0 knee score of 85 points. Panels **a** and **c** are acquired with a sagittal proton-density-weighted turbo spin-echo sequence, whereas panels **b** and **d** are acquired with a coronal proton-density-weighted turbo spin-echo sequence with fat suppression. The white arrows indicate the repair tissue borders

**Fig. 2:**
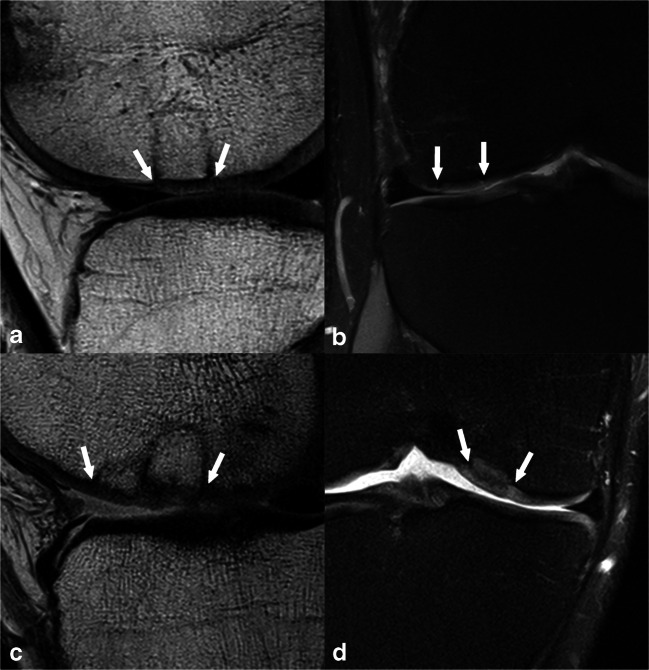
**a**, **b** A 31-year-old male patient 12 months after OAT with complete filling, a split-like integrational defect on the medial transplant border (depicted in image **b**), an intact surface, homogeneous structure, normal signal intensity of the repair tissue, no bony defect or overgrowth, and no subchondral changes, which resulted in an overall MOCART 2.0 knee score of 95 points. Also, the donor region can be appreciated in the anterior medial condyle in image **a**. **c**, **d** A 26-year-old male patient 9 years after OAT with an underfilling of 75–99% of the defect volume, complete integration, an irregular surface < 50% of repair tissue diameter, normal signal intensity, a bony defect ≥ transplant thickness, and no subchondral changes, which resulted in a MOCART 2.0 knee score of 75 points. Panels **a** and **c** are acquired with a sagittal proton-density-weighted turbo spin-echo sequence, whereas panels **b** and **d** are acquired with a coronal proton-density-weighted turbo spin-echo sequence with fat suppression. The white arrows indicate the repair tissue borders

**Fig. 3:**
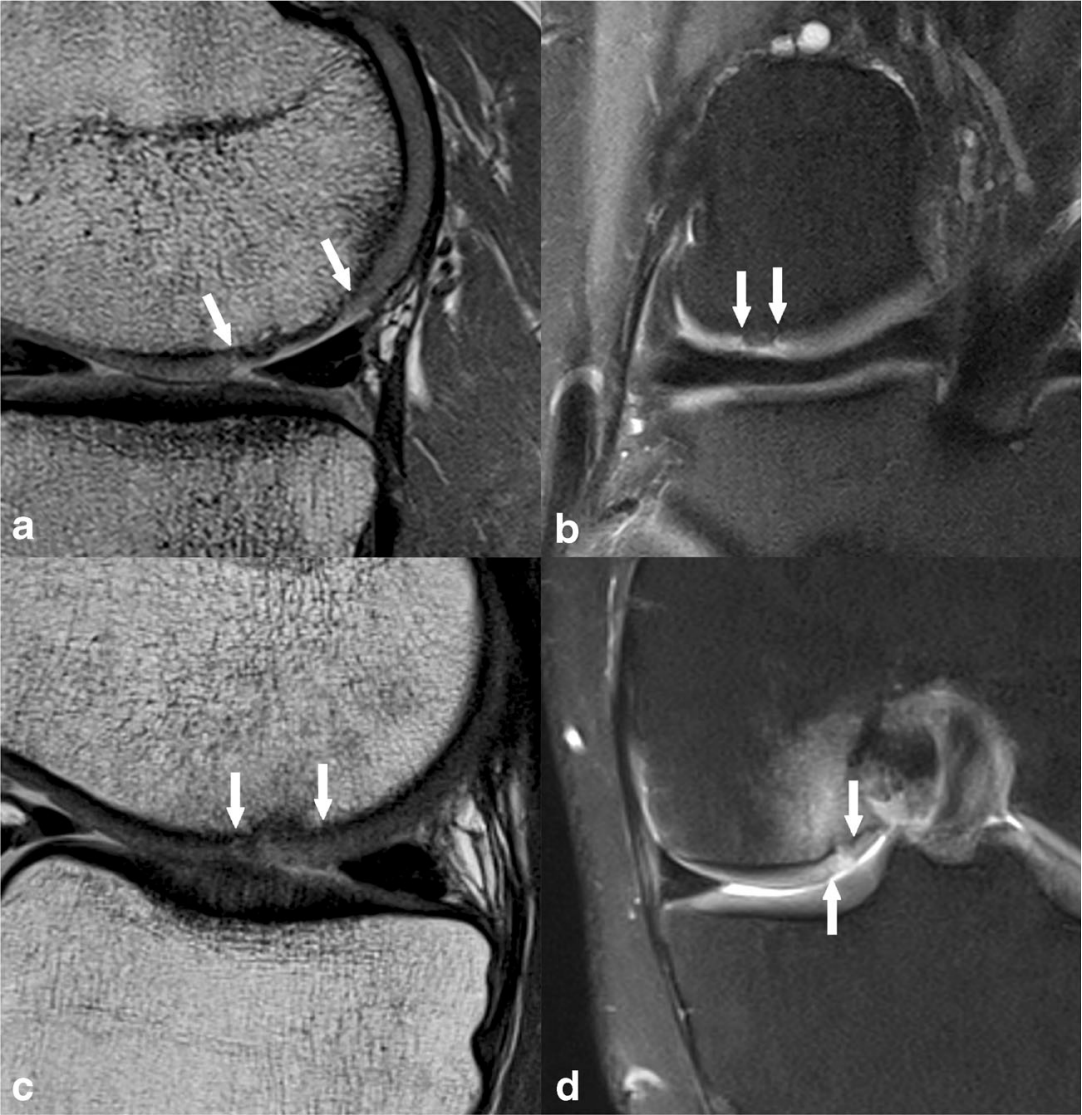
**a**, **b** A 29-year-old male patient 7 months after MFX with an underfilling of 50–74% of the defect volume, complete integration, surface irregularities < 50% of repair tissue diameter, a homogeneous structure, minor hypointense signal intensity, a bony overgrowth < 50% repair tissue thickness, and no subchondral changes, which resulted in a MOCART 2.0 knee score of 60 points. **c**, **d** A 39-year-old male patient 3 months after MFX with a complete filling, complete integration, surface irregularities < 50% of repair tissue diameter, inhomogeneous structure, minor hyperintense signal intensity, a bony defect < 50% repair tissue thickness, and subchondral edema-like signal changes ≥ 50% of the repair tissue diameter, which resulted in a MOCART 2.0 knee score of 75 points. Panels **a** and **c** are acquired with a sagittal proton-density-weighted turbo spin-echo sequence, whereas panels **b** and **d** are acquired with a coronal proton-density-weighted turbo spin-echo sequence with fat suppression. The white arrows indicate the repair tissue borders

One hundred twenty patients, who fit the inclusion criteria, were retrospectively identified. Six patients, who received their follow-up MRI at our institution but had undergone surgery elsewhere, had to be excluded from the analysis due insufficient information regarding the surgical procedure. In total, 114 patients (34 females and 80 males) with a mean age of 32.5 ± 9.6 years at the time of surgery were included. The average defect size was 3.1 ± 2.2 cm^2^. The right knee was affected in 64 patients, whereas the left knee was affected in 50 patients. Seventy-seven defects were located in the medial femoral condyle, 32 in the lateral femoral condyle, and five in the trochlea. The study cohort included 48 patients after ACI, 34 patients after MFX, and 32 patients who were allocated to the osteochondral repair technique group (27 patients after OAT; five patients after MaioRegen^®^, Fin-ceramica). Age at surgery ranged from 30.2 ± 8.8 years in the ACI group to 31.2 ± 10.6 years in the osteochondral repair technique group and to 37.0 ± 8.4 years in the MFX group. The mean postoperative follow-up interval after cartilage repair was 40.5 ± 43.6 months, ranging from 20.2 ± 24.2 months in the MFX group to 35.1 ± 34.6 months in the osteochondral repair technique group and to 57.2 ± 51.7 months in the ACI group. Mean defect size ranged from 2.1 ± 1.0 cm^2^ in the osteochondral repair technique group to 2.1 ± 1.5 cm^2^ in the MFX group and to 4.5 ± 2.5 cm^2^ in the ACI group (Figs. 1, 2, and 3).

For ACI, the following matrices were used in this study population: IGOR.CHONDRO-SYSTEMS^®^ (Institute for Tissue and Organ Reconstruction); Hyalograft C^®^ (Anika Therapeutics); Novocart 3D^®^ (Braun Medical); and CaReS^®^ (Arthro-Kinetics). Additional autologous bone grafting was performed in 16 patients.

Overall, 48 patients were treated for osteochondral lesions (27 OAT cases, five MaioRegen^®^ cases, and 16 ACI with additional autologous bone grafting) and 66 patients were treated for chondral lesions (34 MFX cases and 32 ACI cases).

Age at surgery differed significantly between the ACI and the MFX groups (30.2 ± 8.8 vs. 37.0 ± 8.4 years; *p* = 0.008), but no significant differences were found between the ACI and osteochondral repair technique groups (30.2 ± 8.8 vs. 31.2 ± 10.6 years; *p* = 1.000) or the MFX and osteochondral repair technique groups (*p* = 0.070). The postoperative follow-up interval differed significantly between the ACI and the MFX groups (4.8 ± 4.3 vs. 1.7 ± 2.0 years; *p* = 0.001), but no significant differences were found between the ACI and osteochondral repair technique groups (4.8 ± 4.3 vs. 2.9 ± 2.9 years; *p* = 0.110) or the MFX and osteochondral repair technique groups (*p* = 0.574).

With regard to the defect size, the ACI group (4.5 ± 2.5 cm^2^) differed significantly from the osteochondral repair technique group (2.1 ± 0.98 cm^2^) and the MFX group (2.1 ± 1.5 cm^2^) (both *p* < 0.001) while the osteochondral repair technique group and the MFX group did not differ significantly (*p* = 1.000).

### Interrater and intrarater reliability of the MOCART 2.0 knee score compared to that of the original MOCART score

The overall MOCART 2.0 knee score showed higher interrater reliability than the original MOCART score, with an ICC of 0.875 (95% CI 0.823 to 0.912) versus 0.759 (95% CI 0.660 to 0.831), ranging from 0.863 (95% CI 0.741 to 0.930) in the MFX group to 0.874 (95% CI 0.753 to 0.938) in the osteochondral repair group and to 0.878 (95% CI 0.792 to 0.93) in the ACI group.

Based on the ICC interpretation according to Koo and Li, the majority of the individual variables of the MOCART 2.0 knee score showed substantial agreement with linear-weighted kappa values, ranging from 0.549 (95% CI 0.365 to 0.733) for the variable “surface of the repair tissue” to 0.797 (95% CI 0.713 to 0.882) for the variable “subchondral changes” for all patients.

The intrarater reliability was good, with an overall ICC of 0.866 (95% CI 0.811–0.906) for reader 1 and 0.860 (95% CI 0.803 to 0.901) for reader 2, and showed substantial agreement for the majority of individual variables for all patients. Overall, linear-weighted kappa for reader 1 ranged from 0.463 (95% CI 0.331–0.594) for the variable “surface of the repair tissue” to 0.792 (95% CI 0.705–0.878) for subchondral changes. For reader 2, linear-weighted kappa ranged from 0.585 (95% CI 0.462 to 0.707) for the variable “surface of the repair tissue” to 0.804 (95% CI 0.718 to 0.890) for the variable “subchondral changes” with overlapping 95% confidence intervals for almost all variables and all subgroups.

For a detailed illustration of the interrater and intrarater reliability and a depiction of the agreement for all subgroups, see Tables 2 and 3, respectively.

**Table 2:** Interrater reliability of the original MOCART and MOCART 2.0 knee scores for cartilage repair after ACI versus MFX versus the osteochondral repair technique group (OAT, MaioRegen^®^) given as linear-weighted kappa statistics for individual variables and two-way mixed absolute agreement, single-measure intraclass correlation coefficient (ICC), and their 95% confidence intervals (95% CI) for the resulting total MOCART and MOCART 2.0 knee scores

Interrater reliability of the MOCART and MOCART 2.0 knee score according to repair procedure					
Variables Original MOCART	Interrater agreement Original MOCART		Variables MOCART 2.0 knee score	Interrater agreement MOCART 2.0 knee score	
	Kappa	95% CI		Kappa	95% CI
Overall (*n* = 114)					
Degree	0.620	0.507–0.732	Volume fill	0.697	0.598–0.796
Integration	0.509	0.356–0.662	Integration	0.742	0.623–0.861
Surface	0.467	0.329–0.605	Surface	0.565	0.448–0.682
Structure	0.456	0.289–0.624	Structure	0.595	0.437–0.753
Signal GRE	0.443	0.160–0.707	Signal intensity	0.778	0.674–0.882
Signal T2w	0.589	0.451–0.727			
Subch bone	0.727	0.589–0.865	Bony defect/overgrowth	0.740	0.641–0.839
Subch Lam	0.650	0.513–0.787			
Adhesions	n/a	n/a	Subchondral changes	0.797	0.713–0.882
Synovitis	0.765	0.634–0.896			
	ICC	95% CI		ICC	95% CI
Overall	0.759	0.660–0.831	Overall	0.875	0.823–0.912
ACI (*n* = 48)					
Degree	0.487	0.304–0.670	Volume fill	0.652	0.506–0.798
Integration	0.548	0.322–0.774	Integration	0.719	0.502–0.936
Surface	0.276	0.056–0.495	Surface	0.549	0.365–0.733
Structure	0.429	0.166–0.691	Structure	0.600	0.355–0.845
Signal GRE	0.802	0.545–1.060	Signal intensity	0.783	0.628–0.943
Signal T2w	0.618	0.418–0.818			
Subch bone	0.756	0.560–0.952	Bony defect/overgrowth	0.679	0.508–0.851
Subch Lam	0.500	0.269–0.731			
Adhesions	n/a	n/a	Subchondral changes	0.649	0.480–0.818
Synovitis	0.829	0.645–1.014			
	ICC	95% CI		ICC	95% CI
Overall	0.696	0.492–0.823	Overall	0.878	0.792–0.930
Osteochondral repair technique group (*n* = 32)					
Degree	0.619	0.392–0.846	Volume fill	0.641	0.406–0.876
Integration	0.521	0.245–0.796	Integration	0.670	0.428–0.911
Surface	0.568	0.308–0.828	Surface	0.500	0.286–0.714
Structure	0.491	0.185–0.797	Structure	0.536	0.210–0.862
Signal GRE	0.235	0.005–0.476	Signal intensity	0.901	0.763–1.040
Signal T2w	0.689	0.475–0.903			
Subch bone	0.739	0.462–1.016	Bony defect/overgrowth	0.646	0.398–0.894
Subch Lam	0.688	0.436–0.939			
Adhesions	n/a	n/a	Subchondral changes	0.848	0.714–0.983
Synovitis	0.788	0.560–1.015			
	ICC	95% CI		ICC	95% CI
Overall	0.706	0.476–0.845	Overall	0.874	0.753–0.938
MFX (*n* = 34)					
Degree	0.769	0.613–0.925	Volume fill	0.788	0.636–0.940
Integration	0.462	0.178–0.747	Integration	0.826	0.664–0.987
Surface	0.627	0.414–0.841	Surface	0.667	0.473–0.861
Structure	0.448	0.141–0.754	Structure	0.628	0.361–0.895
Signal GRE	0.233	− 0.399 to 0.864	Signal intensity	0.659	0.441–0.878
Signal T2w	0.428	0.125–0.732			
Subch bone	0.731	0.485–0.977	Bony defect/overgrowth	0.832	0.675–0.989
Subch Lam	0.822	0.631–1.014			
Adhesions	n/a	n/a	Subchondral changes	0.918	0.824–1.011
Synovitis	0.656	0.381–0.931			
	ICC	95% CI		ICC	95% CI
Overall	0.870	0.746–0.934	Overall	0.863	0.741–0.930

**Table 3:** Intrarater reliability of the original MOCART and MOCART 2.0 knee scores for cartilage repair after ACI versus MFX versus the osteochondral repair technique group (OAT, MaioRegen^®^) given as linear-weighted kappa statistics for individual variables and two-way mixed absolute agreement, single-measure intraclass correlation coefficients (ICCs), and their 95% confidence intervals (95% CI) for the resulting total MOCART and MOCART 2.0 knee scores for both readers

Intrarater reliability of the original MOCART and MOCART 2.0 knee score according to repair procedure									
Variables Original MOCART	Intrarater agreement Reader 1 Original MOCART		Intrarater agreement Reader 2 Original MOCART		Variables MOCART 2.0 knee score	Intrarater agreement Reader 1 MOCART 2.0 knee score		Intrarater agreement Reader 2 MOCART 2.0 knee score	
	Kappa	95% CI	Kappa	95% CI		Kappa	95% CI	Kappa	95% CI
Overall (*n* = 114)									
Degree	0.644	0.549–0.740	0.611	0.510–0.711	Volume fill	0.737	0.641–0.834	0.696	0.595–0.797
Integration	0.684	0.576–0.792	0.553	0.405–0.701	Integration	0.707	0.577–0.838	0.742	0.633–0.850
Surface	0.392	0.265–0.520	0.552	0.432–0.671	Surface	0.463	0.331–0.594	0.585	0.462–0.707
Structure	0.677	0.523–0.832	0.484	0.317–0.652	Structure	0.695	0.553–0.836	0.714	0.575–0.853
Signal GRE	0.732	0.544–0.921	0.686	0.473–0.900	Signal	0.786	0.687–0.885	0.802	0.711–0.893
Signal T2w	0.445	0.299–0.592	0.531	0.387–0.675					
Subch bone	0.703	0.553–0.853	0.638	0.477–0.800	Bony defect/overgrowth	0.713	0.614–0.812	0.770	0.674–0.866
Subch Lam	0.703	0.577–0.829	0.578	0.430–0.725					
Adhesions	1.000		1.000		Subchondral changes	0.792	0.705–0.878	0.804	0.718–0.890
Synovitis	0.730	0.594–0.867	0.690	0.540–0.840					
	ICC	95% CI	ICC	95% CI		ICC	95% CI	ICC	95% CI
Overall	0.759	0.522–0.866	0.767	0.592–0.859	Overall	0.866	0.811–0.906	0.860	0.803–0.901
ACI (*n* = 48)									
Degree	0.598	0.461–0.735	0.599	0.427–0.771	Volume fill	0.704	0.597–0.902	0.595	0.417–0.772
Integration	0.701	0.547–0.854	0.478	0.218–0.739	Integration	0.699	0.478–0.921	0.625	0.405–0.844
Surface	0.432	0.250–0.615	0.482	0.310–0.654	Surface	0.432	0.225–0.638	0.489	0.288–0.691
Structure	0.591	0.337–0.844	0.595	0.368–0.822	Structure	0.542	0.286–0.798	0.744	0.534–0.953
Signal GRE	0.650	0.338–0.963	0.705	0.395–1.015	Signal	0.777	0.631–0.923	0.797	0.651–0.943
Signal T2w	0.471	0.252–0.690	0.457	0.234–0.678					
Subch bone	0.707	0.488–0.925	0.626	0.392–0.860	Bony defect/overgrowth	0.649	0.485–0.813	0.801	0.660–0.941
Subch Lam	0.629	0.436–0.821	0.581	0.364–0.797					
Adhesions	1.000		1.000		Subchondral changes	0.650	0.480–0.819	0.920	0.839–1.000
Synovitis	0.614	0.362–0.866	0.416	0.089–0.743					
	ICC	95% CI	ICC	95% CI		ICC	95% CI	ICC	95% CI
Overall	0.763	0.524–0.875	0.741	0.481–0.864	Overall	0.871	0.780–0.925	0.848	0.745–0.912
Osteochondral repair technique group (*n* = 32)									
Degree	0.631	0.403–0.860	0.581	0.374–0.788	Volume fill	0.848	0.721–0.974	0.713	0.516–0.911
Integration	0.681	0.501–0.862	0.631	0.387–0.875	Integration	0.688	0.454–0.922	0.642	0.420–0.863
Surface	0.216	− 0.019 to 0.452	0.683	0.460–0.906	Surface	0.407	0.157–0.658	0.662	0.440–0.884
Structure	0.517	0.111–0.923	0.417	0.065–0.769	Structure	0.925	0.781–1.069	0.626	0.330–0.922
Signal GRE	0.930	0.816–1.044	0.494	0.005–0.982	Signal	0.799	0.621–0.977	0.817	0.655–0.979
Signal T2w	0.463	0.191–0.735	0.666	0.392–0.940					
Subch bone	0.604	0.199–1.008	0.632	0.174–1.089	Bony defect/overgrowth	0.690	0.478–0.902	0.767	0.573–0.960
Subch Lam	0.772	0.529–1.015	0.571	0.271–0.872					
Adhesions	1.000		1.000		Subchondral changes	0.787	0.627–0.947	0.631	0.418–0.844
Synovitis	0.772	0.529–1.015	0.710	0.450–0.970					
	ICC	95% CI	ICC	95% CI		ICC	95% CI	ICC	95% CI
Overall	0.765	0.469–0.894	0.824	0.658–0.914	Overall	0.912	0.824–0.957	0.857	0.727–0.928
MFX (*n* = 34)									
Degree	0.671	0.489–0.853	0.620	0.463–0.777	Degree	0.628	0.432 0.825	0.754	0.592–0.915
Integration	0.660	0.420–0.900	0.558	0.293–0.823	Integration	0.715	0.484–0.946	0.929	0.835–1.022
Surface	0.468	0.231–0.706	0.532	0.300–0.764	Surface	0.577	0.373–0.780	0.598	0.366–0.831
Structure	0.672	0.408–0.935	0.377	0.058–0.697	Structure	0.686	0.438–0.935	0.742	0.506–0.979
Signal GRE	0.571	0.082–1.061	0.571	0.082–1.061	Signal	0.785	0.590–0.979	0.814	0.653–0.975
Signal T2w	0.393	0.119–0.666	0.517	0.257–0.776					
Subch bone	0.735	0.501–0.970	0.619	0.353–0.886	Bony defect/overgrowth	0.754	0.576–0.931	0.710	0.526–0.893
Subch Lam	0.679	0.400–0.959	0.494	0.176–0.811					
Adhesions	1.000		1.000		Subchondral changes	0.973	0.919–1.026	0.831	0.693–0.969
Synovitis	0.810	0.606–1.015	0.866	0.685–1.046					
	ICC	95% CI	ICC	95% CI		ICC	95% CI	ICC	95% CI
Overall	0.758	0.423–0.891	0.770	0.473–0.894	Overall	0.812	0.612–0.907	0.864	0.732–0.931

### Inter- and intrarater reliability of the MOCART 2.0 knee score for chondral versus osteochondral cartilage lesions

Overall, the interrater reliability was higher for osteochondral lesions than for chondral lesions with ICCs of 0.906 (95% CI 0.838 to 0.947) for osteochondral lesions versus 0.786 (95% CI 0.674 to 0.863) for chondral lesions, respectively. The same was true for the intrarater reliability for both readers with ICCs of 0.921 (95% CI 0.862–0.955) and 0.869 (95% CI 0.778–0.924) for osteochondral lesions versus 0.804 (95% CI 0.699–0.876) and 0.839 (95% CI 0.736–0.902) for chondral lesions.

For chondral lesions, linear-weighted kappa statistics showed mostly substantial agreement for both interrater and intrarater agreements. Interrater agreement ranged from 0.588 (95% CI 0.428 to 0.747) for the variable “surface of the repair tissue” to 0.821 (95% CI 0.711 to 0.930) for the variable “subchondral changes.” Intrarater agreement ranged from linear-weighted kappa values of 0.486 (95% CI 0.321 to 0.651) for reader 1 for the variable “surface of the repair tissue” to 0.861 (95% CI 0.771 to 0.952) for reader 2 for the variable “subchondral changes.”

For osteochondral lesions, linear-weighted kappa statistics showed mostly substantial agreement for both interrater and intrarater agreements as well. Interrater agreement ranged from 0.534 (95% CI 0.362 to 0.706) for the variable “surface” to 0.891 (95% CI 0.782 to 1.000) for the variable “signal intensity.” Intrarater agreement ranged from 0.435 (95% CI 0.224 to 0.646) for the variable “surface of the repair tissue” for reader 1 to 0.871 (95% CI 0.777 to 0.966) for reader 1 for the variable “volume of cartilage defect filling compared to native cartilage” (for a detailed overview, see Table 4).

**Table 4:** Interrater and intrarater reliability of the MOCART 2.0 knee score for cartilage repair after chondral (MFX and ACI) versus osteochondral lesions (OAT, MaioRegen^®^, or ACI with autologous bone grafting) given as linear-weighted kappa statistics for individual variables and two-way mixed absolute agreement, single-measure intraclass correlation coefficients (ICCs), and their 95% confidence intervals (95% CI) for the resulting total MOCART 2.0 knee score

Interrater and intrarater reliability of the MOCART 2.0 knee score according to lesion type (chondral vs. osteochondral lesions)						
Variables MOCART 2.0 knee score	Interrater reliability		Intrarater reliability Reader 1		Intrarater reliability Reader 2	
	Kappa	95% CI	Kappa	95% CI	Kappa	95% CI
Chondral lesions (*n* = 66)						
Volume fill	0.698	0.571–0.825	0.647	0.503–0.791	0.684	0.548–0.820
Integration	0.686	0.502–0.869	0.759	0.611–0.908	0.662	0.448–0.876
Surface	0.588	0.428–0.747	0.486	0.321–0.651	0.564	0.400–0.728
Structure	0.603	0.405–0.801	0.634	0.442–0.825	0.818	0.665–0.971
Signal intensity	0.691	0.538–0.844	0.795	0.663–0.927	0.781	0.654–0.907
Bony defect/overgrowth	0.768	0.649–0.887	0.665	0.532–0.798	0.751	0.623–0.879
Subchondral changes	0.821	0.711–0.930	0.826	0.717–0.935	0.861	0.771–0.952
	ICC	95% CI	ICC	95% CI	ICC	95% CI
Overall	0.786	0.674–0.863	0.804	0.699–0.876	0.839	0.736–0.902
Osteochondral lesions (*n* = 48)						
Volume fill	0.694	0.535–0.852	0.871	0.777–0.966	0.673	0.505–0.842
Integration	0.783	0.627–0.940	0.741	0.579–0.903	0.687	0.512–0.862
Surface	0.534	0.362–0.706	0.435	0.224–0.646	0.600	0.410–0.789
Structure	0.578	0.317–0.839	0.789	0.592–0.986	0.576	0.336–0.817
Signal intensity	0.891	0.782–1.000	0.771	0.662–0.920	0.846	0.721–0.970
Bony defect/overgrowth	0.687	0.509–0.864	0.774	0.625–0.924	0.819	0.679–0.958
Subchondral changes	0.761	0.626–0.896	0.742	0.602–0.881	0.728	0.570–0.886
	ICC	95% CI	ICC	95% CI	ICC	95% CI
Overall	0.906	0.838–0.947	0.921	0.862–0.955	0.869	0.778–0.924

## Discussion

The aim of this study was to evaluate the reliability of the recently introduced MOCART 2.0 knee score for the assessment of the radiological outcome after different cartilage repair procedures and in different lesion types.

The main finding of this study is that the ICCs of the total resultant MOCART 2.0 knee score showed good or excellent agreement, regardless of treatment modality. Furthermore, the majority of the categorical variables of the MOCART 2.0 knee score showed substantial agreement in the inter- and intrarater reliability testing, again regardless of treatment modality. When compared to the original MOCART score, higher interrater reliability was observed for almost all individual variables and the overall scoring, independent of surgical treatment (Table 2). The same was true when comparing the intrarater reliability between the original MOCART score and the MOCART 2.0 knee score (Table 3). This difference might be attributed in part to the modification of variables as well as to the atlas that was introduced alongside the MOCART 2.0 knee score, which has demonstrated to positively impact the reliability of less-experienced readers [22].

Interestingly, whereas overall interrater reliability was higher for osteochondral lesions than for chondral lesions, the individual variables “bony defect” and “subchondral changes” demonstrated worse interrater reliability for osteochondral lesions. We attribute this finding to the overall higher number of pathological findings in the osteochondral lesion group with regard to these two variables.

Since its introduction, a handful of studies have employed the MOCART 2.0. knee score for the assessment of radiological outcome after all arthroscopic matrix-encapsulated autologous chondrocyte implantation [27] or MACI [28] in the knee and AMIC in the treatment of osteochondral lesions of the talus [29]. However, none of these studies assessed intra- or interrater reliability. Casari et al [29] employed the original MOCART score and the MOCART 2.0. knee score for the assessment of AMIC in the repair or osteochondral lesions of the talus. Whereas they did not assess intra- or interrater reliability, they found a significant correlation between preoperative lesion size and postoperative MOCART scores, but no correlation with clinical outcome. When interpreting these results, it has to be considered that low inter- and intrarater reliability has been reported for the assessment of cartilage repair of the talus with the original MOCART score [30].

For individual variables, the reproducibility was consistently lowest in this study for “surface of the repair tissue.” This was true for the overall population, as well as most subpopulations for the original MOCART score as well as for the MOCART 2.0 knee score. Interestingly, this was not the case in the first publication on interrater variability of the original MOCART score [16]. We attribute this finding partly to the increased quality and resolution of the imaging protocol, when compared to the study of Marlovits et al [16], which employed a 1-T system for imaging. Whereas high-resolution imaging is deemed necessary to visualize discrete fissuring, it is not necessarily associated with increased reliability, as minor fissures, which would have been underappreciated by a lower resolution sequence, might be seen by one reader but not the other.

For most variables of the MOCART 2.0 knee score, the inter- and intrarater reliabilities observed in this study were slightly lower than the inter- and intrarater reliabilities for the expert readers, but higher than the inter- and intrarater reliabilities for the inexperienced readers in the study that first introduced the MOCART 2.0 knee score [22]. This might be due to the fact that, while being residents, both readers specialized in the morphological and quantitative assessment of knee MRIs and supports the interpretation of the results of this study. Because even if the intra- and interrater reproducibility for these readers were significantly lower than for experienced readers, it can be assumed that it would be similarly lower regardless of the type of lesion or surgical treatment strategy. Furthermore, inter- and intrarater reliabilities for most variables of the MOCART 2.0 knee score reported in this study demonstrated less variability than previously reported [22]. This might be mainly attributed to the higher number of patients in this current study.

Interrater reliability of the individual ordinal variables of the original MOCART score was consistently lower in our study than previously reported [16]. However, in addition to employing expert readers, ICCs were used for the assessment of reliability of the individual ordinal variables. Whereas the use of linear-weighted kappa values is more adequate for ordinal variables, it is more rigorous.

No adhesions were observed in the study cohort, which corroborated the obsolescence of the variable “adhesions” in the original MOCART score and its consequential removal in the MOCART 2.0 knee score.

There are several limitations in this study that have to be mentioned.

A direct numerical comparison of the overall MOCART 2.0 knee score, as well as its categorical variables between treatment groups, and also chondral and osteochondral defects, would be highly interesting. However, considering the limitations of the retrospective design of the study, this would be a biased comparison. Due to the retrospective nature of the study, patients were not randomly allocated to different surgical treatment strategies. Treatment decisions were rather based on demographics and disease-specific factors. Hence, there were systematic differences regarding age, as well as lesion size between the different repair techniques. These differences are to be expected when retrospectively evaluating a cohort that was allocated to their respective treatment based on current guidelines [31].

However, even though the defect size in the ACI group was significantly different than in the OAT and MFX groups, size did not show a significant correlation with the resultant MOCART 2.0 knee score.

Also, sequence parameters of the MRI examinations differed slightly between patients, since they were part of the clinical routine on different scanners. However, all included imaging studies were conducted on 3-T systems with dedicated knee coils. Furthermore, with 48 ACI patients, 32 patients in the osteochondral repair technique group, and 34 MFX patients, group sizes were unequally distributed. However, for the assessment of inter- and interrater reliability, the number of patients seemed to be adequate.

This study demonstrates comparable intra- and interrater reliability of the MOCART 2.0 knee score for the radiological assessment of different cartilage repair techniques (ACI vs. osteochondral repair techniques vs. MFX), as well as for the treatment of chondral versus osteochondral defects. To allow for a comparison of the absolute values of the MOCART 2.0 knee score between different repair techniques, a matched pair analysis or a randomized trial would be necessary. For the identification of a potential correlation with clinical outcome or even more importantly a potential predictive value regarding clinical outcome, additional longitudinal studies and correlation with clinical data would be necessary.

## Abbreviations

ACI: Autologous chondrocyte implantation
BMS: Bone marrow stimulation
95% CI: 95% confidence interval
Fs: Fat-saturated
ICC: Intraclass correlation coefficient
MFX: Microfracture
MOCART: Magnetic Resonance Observation of Cartilage Repair Tissue
MRI: Magnetic resonance imaging
OAT: Osteochondral autologous transplantation
PDw: Proton-density-weighted
T1w: T1-weighted
TSE: Turbo spin-echo
